# Supplementary material for: Effects of Chinese Martial Arts on Motor Skills in Children between 5 and 6 Years of Age: A Randomized Controlled Trial
Source: Int J Environ Res Public Health. 2022 Aug 17;19(16):10204. doi: 10.3390/ijerph191610204 (PMC9408615; doi:10.3390/ijerph191610204)

Attachment 1.

1.Illustration of teaching intervention contents

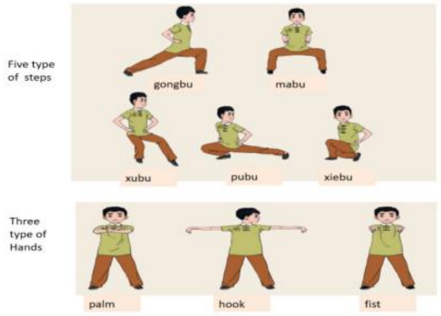

1-4 lessons

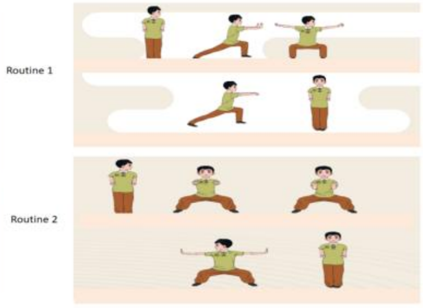

5-8 lessons

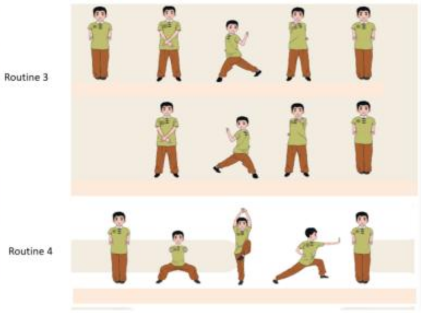

9-12 lessons

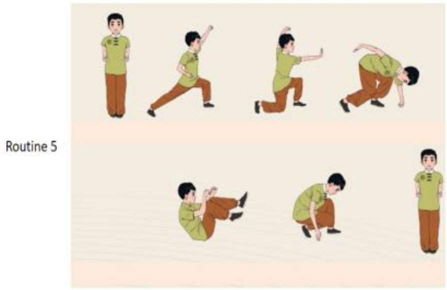

13-14 lessons

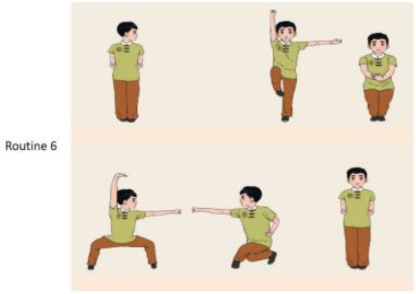

15-16 lessons

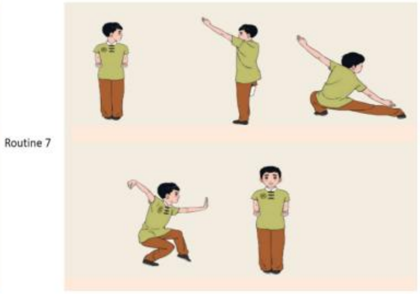

16-18 lessons

2. Weekly teaching schedule

Weeks	contents	tasks	Learning time (Mins)/frequency
1	three types of hand	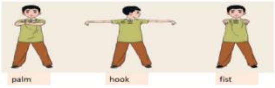	30/1
1-2	Five types of step	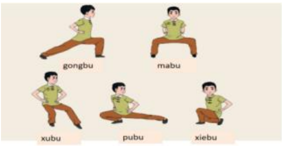	90/3
3	Routine 1	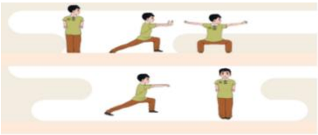	60/2
4	Routine 2	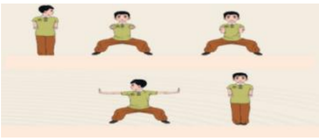	60/2
5	Routine 3	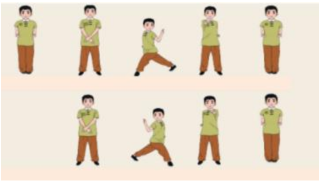	60/2
6	Routine 4	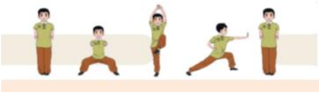	60/2
7	Routine 5	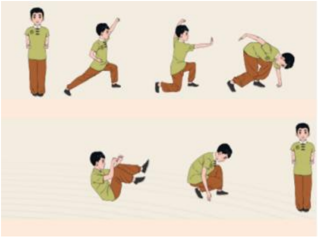	60/2
8	Routine 6	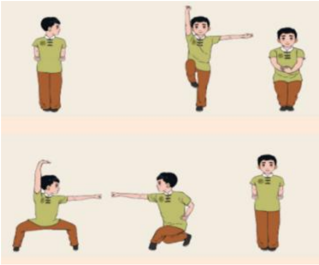	60/2

9	Routine 7	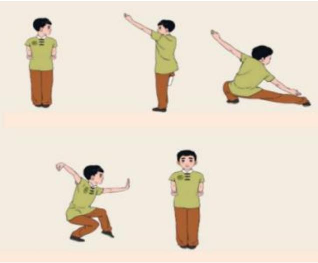	60/2
10	Training consolidation	Above all	60/2

3.teaching case

Teaching case of Mabu	
MAST	MATT
teaching procedure:<br>Make Mabu into life(activate old knowledge)-multi-sensory interaction(form a concept of Mabu)--Gamification experience(dynamic stereotype)--Group competition(invigorate health effectively)	teaching procedure:<br>Introduce teaching contents--Teacher explanation and demonstration--Students imitate learning--group exercises--Concentrated consolidation exercises
**Make Mabu into life:**<br>Connect the Mabu with the action of horse riding, watch the pictures of horse riding, and summarize the main points of the action: body upright, feet apart, hips and knees flexed.<br>**multi-sensory interaction:**<br>1)Watch the video to intuitively perceive the movements of horse riding. Children initially imitate the movements of horse riding, requiring ups and downs to practice the flexibility of the hip and knee joints.<br>2)Step on a parallel line to imitate horse riding (the distance between the two lines is about 25cm), and practice jumping and jumping with one foot on the parallel line to become a Mabu(the two feet must always be on the parallel line), improves muscle proprioception in the span of the feet.<br>3)Holding a martial arts stick in front of the body perpendicular to the ground to practice Mabu, through the interactive fusion of vision and proprioceptive sense, to ensure the integrity of the body, and to strengthen the sense of distance between the two feet in the process of turning left and right.<br>4)Practice the Mabu by placing the stick on your thigh and keeping the stick from slipping off, make sure your thighs are parallel to the ground.<br>**Gamification experience:**<br>Game 1, in pairs, standing face to face, one slaps the other child on the shoulder with both hands, and the other child quickly makes a Mabu to avoid being slapped.	**Introduce teaching contents:**<br>Introduce the task, requirements and goals to the children before class.<br>**Teacher explanation and demonstration:**<br>demonstrates the Mabu, explaining the requirements of the movement, that is feet open, body upright, knees and hips flexed.<br>**Students imitate learning:**<br>The teacher breaks down the movements. First, tell the children to measure the distance between the three feet. Second, slowly bend their knees and bend their hips to squat. Third, tell the children that their bodies must remain upright. The teacher demonstrates while explaining, and the children imitate step by step until they basically learned.<br>**group exercises:**<br>Divide into groups, arrange backbones to lead, practice consolidating movements, and teachers make rounds to correct movements.<br>**Concentrated consolidation exercises:**<br>After the teacher's inspection, summarize the main problems in the exercise, focus on explaining, and then focus on practicing to improve and consolidate.

Game 2,in pairs, one child presses the shoulders of the other child practicing Mabu with both hands, and the other child exerts force to keep the Mabu unchanged, and the body does not sway.<br>Game 3,two children stand on the same straight line and do Mabu facing each other. The adjacent feet are close together. The two children pull each other on the same side. Whoever can keep it longer wins.<br>**Group competition:**<br>Divide the children into six equal groups, each with an inflatable rubber horse, and practice the relay race between two lines separated by a distance of 10M. The group with the shortest time is the winner and can get a reward (small gift).	

4.the record of teaching moment(DR. Li is teaching)

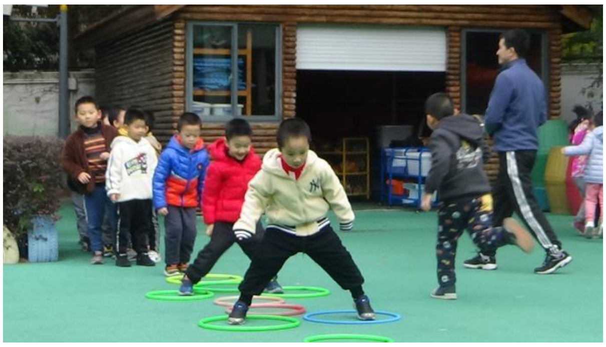

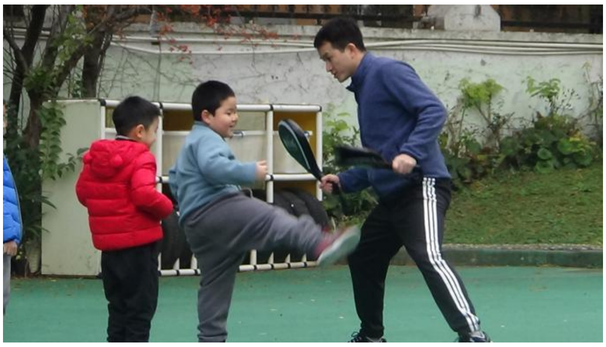

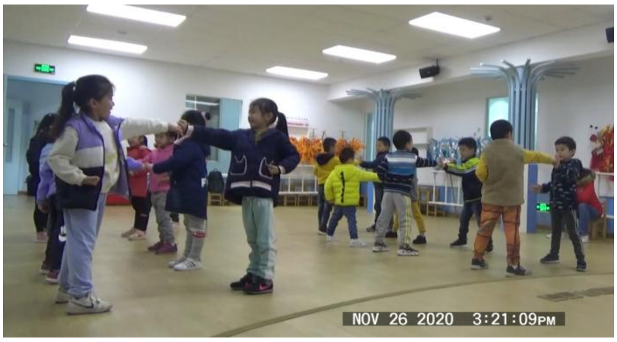

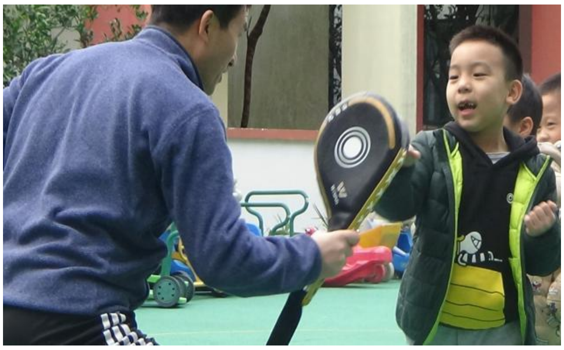

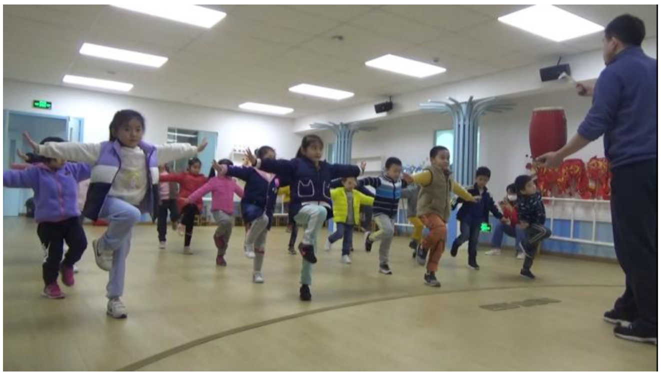

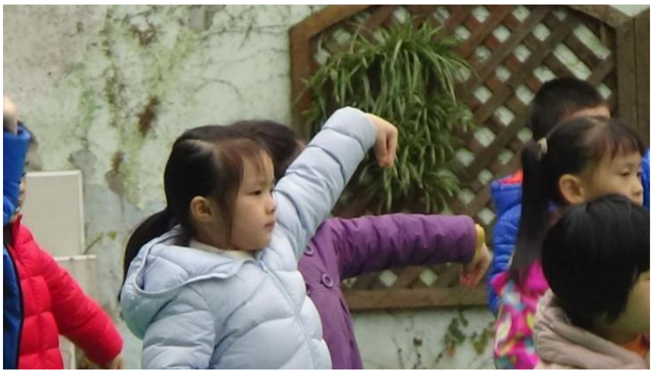

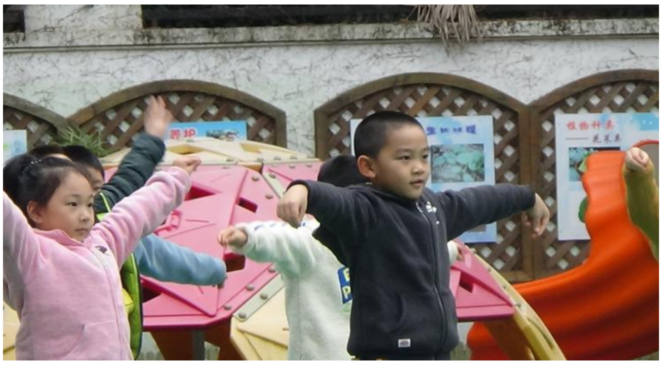

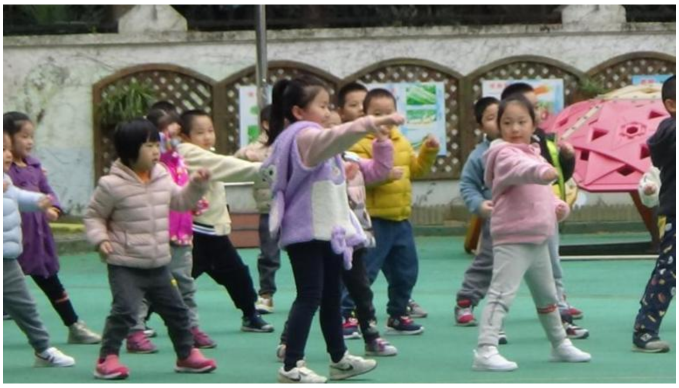

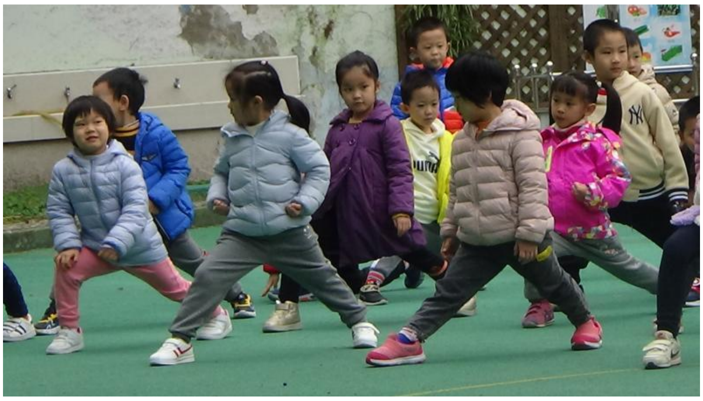

Attachment 2.

Test training for the graduated students:

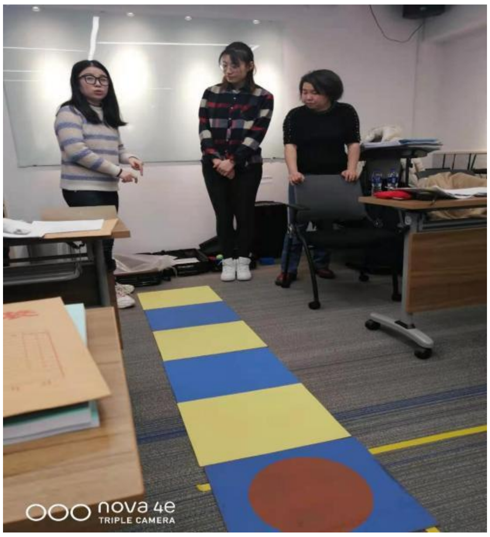

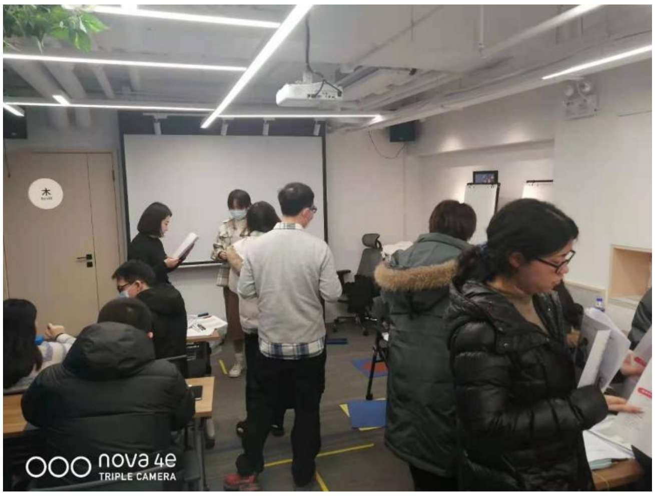

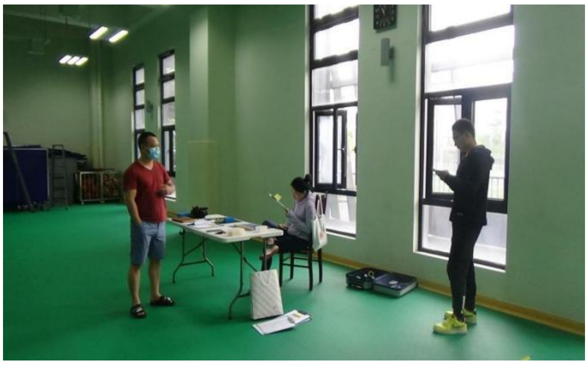

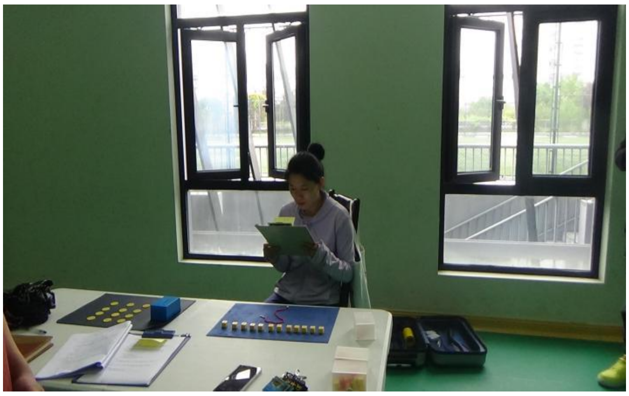

Test site:

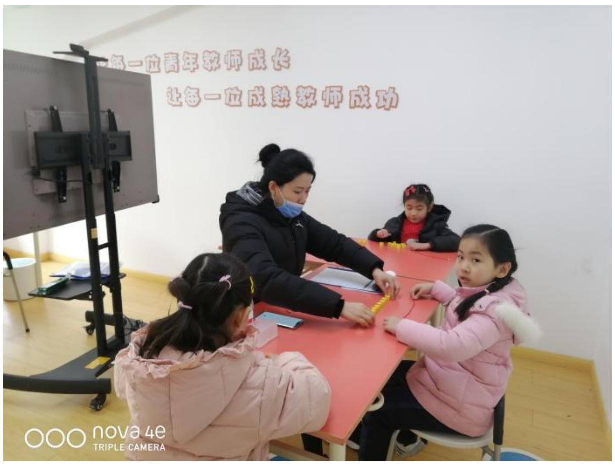

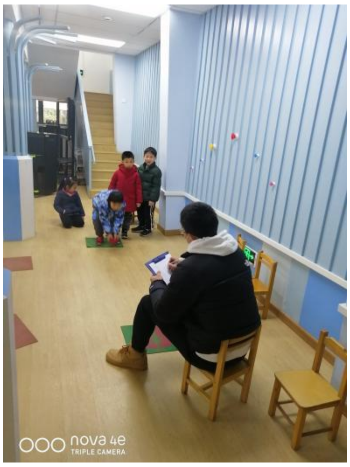

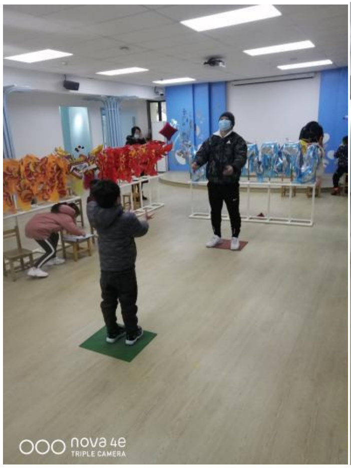

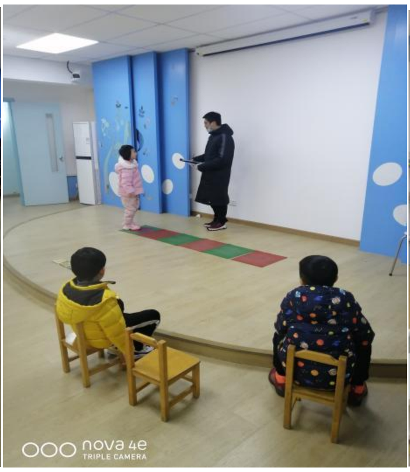

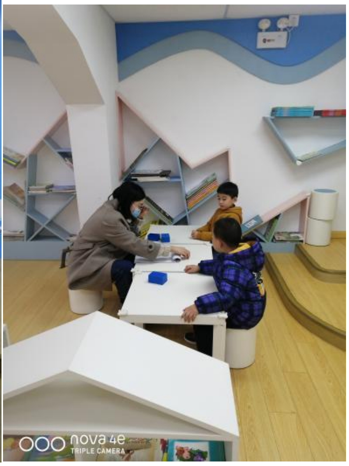

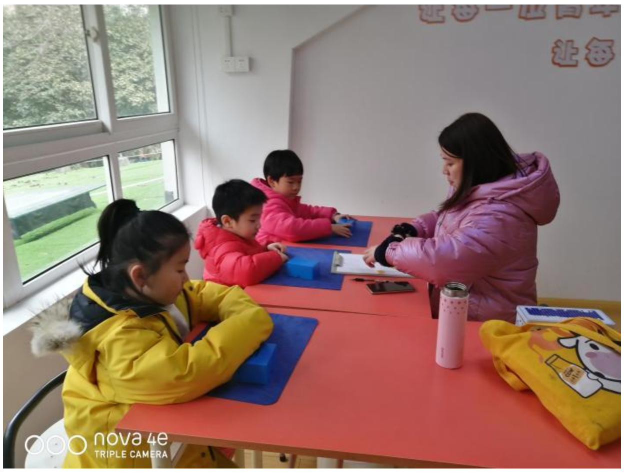

Test team(6 researchers of 8):

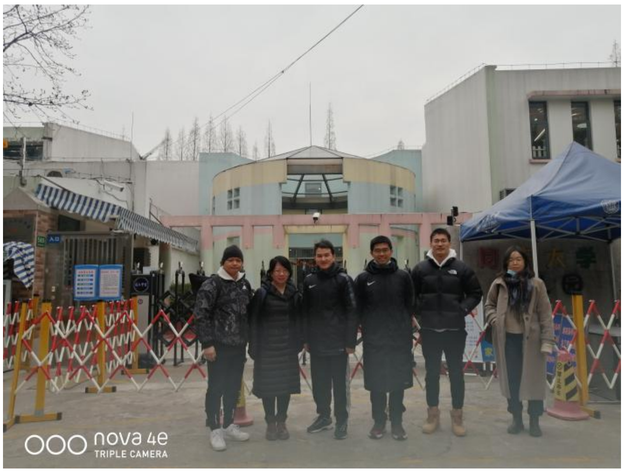

Supplement: Supplementary file 1 [file ijerph-19-10204-s001.zip › ijerph-1843647-supplementary.pdf]
